# Advances of deep learning in electrical impedance tomography image reconstruction

**DOI:** 10.3389/fbioe.2022.1019531

**Published:** 2022-12-14

**Authors:** Tao Zhang, Xiang Tian, XueChao Liu, JianAn Ye, Feng Fu, XueTao Shi, RuiGang Liu, CanHua Xu

**Affiliations:** ^1^ Department of Biomedical Engineering, The Fourth Military Medical University, Xi’an, China; ^2^ Shaanxi Key Laboratory for Bioelectromagnetic Detection and Intelligent Perception, Xi’an, China; ^3^ Drug and Instrument Supervision and Inspection Station, Xining Joint Logistics Support Center, Lanzhou, China

**Keywords:** electrical impedance tomography, deep learning, image reconstruction, medical imaging, research progress

## Abstract

Electrical impedance tomography (EIT) has been widely used in biomedical research because of its advantages of real-time imaging and nature of being non-invasive and radiation-free. Additionally, it can reconstruct the distribution or changes in electrical properties in the sensing area. Recently, with the significant advancements in the use of deep learning in intelligent medical imaging, EIT image reconstruction based on deep learning has received considerable attention. This study introduces the basic principles of EIT and summarizes the application progress of deep learning in EIT image reconstruction with regards to three aspects: a single network reconstruction, deep learning combined with traditional algorithm reconstruction, and multiple network hybrid reconstruction. In future, optimizing the datasets may be the main challenge in applying deep learning for EIT image reconstruction. Adopting a better network structure, focusing on the joint reconstruction of EIT and traditional algorithms, and using multimodal deep learning-based EIT may be the solution to existing problems. In general, deep learning offers a fresh approach for improving the performance of EIT image reconstruction and could be the foundation for building an intelligent integrated EIT diagnostic system in the future.

## 1 Introduction

Electrical impedance tomography (EIT) is a non-invasive imaging method for reconstructing the distribution or changes in electrical properties by applying a safe alternating current excitation, measuring the surface voltage signal, and using a reconstruction algorithm. With the advantages of being radiation-free and inexpensive, and allowing real-time imaging, it has been extensively utilized in geophysical imaging, multiphase flow monitoring, and biomedical imaging (Menden et al., 2021a; Menden et al., 2021b; Hsu et al., 2021; Jiang et al., 2021). In terms of medical applications, EIT is regarded as a functional imaging method compared with traditional computed tomography (CT) and ultrasound, which reflect the pathophysiological information of the human body through impedance changes. Presently, it shows good potential for application in numerous clinical settings. For example, Draeger Medical Devices has developed the first commercial EIT equipment for pulmonary function monitoring and conducted several clinical studies (Bickenbach et al., 2017; Eronia et al., 2017; Longhini et al., 2019; Zhao et al., 2019). Particularly during the COVID-19 pandemic, EIT has provided a potential reference for the decision-making of patients' treatment (Hsu et al., 2021; Nascimento et al., 2021; Bayford et al., 2022), thereby demonstrating the significant clinical application value of pulmonary EIT in the management of patients suffering from severe respiratory failure (Zhao et al., 2020; Bronco et al., 2021). In the case of brain injury monitoring, Fu et al. demonstrated the important role of EIT in the treatment of mannitol dehydration (Fu et al., 2014). Subsequently, Yang et al. first studied the comparative relationship between EIT and intracranial pressure and confirmed that EIT could also be used to track changes related to cerebral edema (Yang et al., 2019). Moreover, they performed extensive research on hardware systems and algorithms for EIT clinical application (Shi et al., 2018; Liu et al., 2019; Li et al., 2019; Ma et al., 2019; Li et al., 2020). In contrast, Holder et al. used EIT for the first time to locate epileptic lesions in animal experiments (Boone et al., 1994), and monitored different physiological changes between seizures and during epileptic activity (Hannan et al., 2018). They achieved deep neural activity imaging (Faulkner et al., 2018) and imaging of the hippocampus (Hannan et al., 2021). Furthermore, they confirmed that the use of electroencephalogram (EEG) combined with EIT monitoring improved the diagnosis rate of epilepsy patients (Witkowska-Wrobel et al., 2021). Recently, the Holder group made good progress in rapid neural network EIT (Aristovich et al., 2018; Ravagli et al., 2020). In addition, EIT has shown good application prospects in other types of brain imaging, such as brain stroke detection (Romsauerova et al., 2006; Yang et al., 2016; Goren et al., 2018), brain tumor detection (Meng et al., 2013), and brain abscess (Oh et al., 2013; Kim et al., 2015). For breast cancer detection using EIT, a variety of equipment has been developed and clinical studies have been conducted (Kao et al., 2008; Ji et al., 2009; Zhang et al., 2014). Furthermore, You et al. first reported the case of retroperitoneal hemorrhage EIT monitoring in patients with renal trauma (You et al., 2013), and Liu et al. reported the first study on non-invasive monitoring of cerebral blood volume during total aortic arch replacement (Liu et al., 2019). In addition to the abovementioned fields, EIT has extensive applications in biomedical areas, such as cell culture monitoring (Yang et al., 2019; Schwarz et al., 2020; Liu et al., 2022b) and bioimpedance analysis (Cortesi et al., 2021). These studies fully demonstrate that EIT, as a new medical imaging technology, is gradually becoming a powerful supplement to traditional medical imaging technology.

Most of the time, image reconstruction is one of the main concerns of EIT researchers. Image reconstruction methods of EIT can be divided into time-difference, frequency-difference, and absolute imaging. Time-difference imaging, also known as dynamic imaging, uses the measurement data at different times to obtain images of changes in conductivity distribution through differential imaging algorithms (Zhang et al., 2021). Frequency-difference imaging is based on the difference in spectral characteristics between biological tissues, wherein a reconstructed image is obtained by applying excitation currents of different frequencies using a multi-frequency imaging algorithm (McDermott et al., 2020). Absolute imaging, also known as static imaging, uses measurement data at a specific moment to obtain the distribution of conductivity through an inverse problem reconstruction algorithm (Hamilton et al., 2018). Owing to the severely ill-posed and ill-posed nature of the EIT inverse problem, static imaging is very sensitive to noise and boundary conditions, and obtaining imaging results suitable for clinical applications stably is difficult. To date, time-difference imaging has been used primarily in clinical research studies. However, the imaging results are easily affected by noise, thereby resulting in low spatial resolution. Thus, exploring new EIT reconstruction algorithms to improve image quality has attracted considerable attention.

With the development of deep learning in natural language processing, speech recognition, image processing, computer vision and other fields, more researchers focus on its application in medical image reconstruction, such as reconstruction of low-dose CT and fast magnetic resonance imaging (MRI) (Kim et al., 2019; Ben Yedder et al., 2020; Anaya-Isaza et al., 2021). Considering the advantages of applying deep learning in image reconstruction, some researchers have applied deep learning in EIT reconstruction to enhance image quality and spatial resolution.

This study systematically reviews the application progress of deep learning in EIT image reconstruction, focusing on direct reconstruction of a single neural network, joint reconstruction of traditional algorithms and deep learning methods, and hybrid reconstruction of multiple deep neural networks (DNNs). The results indicate that the traditional algorithm combined with deep learning reconstruction and a variety of DNN hybrid reconstruction strategies have greater advantages compared with traditional algorithms in EIT image reconstruction, and great potential for future clinical applications.

## 2 Basic principles of EIT

**FIGURE 1:**
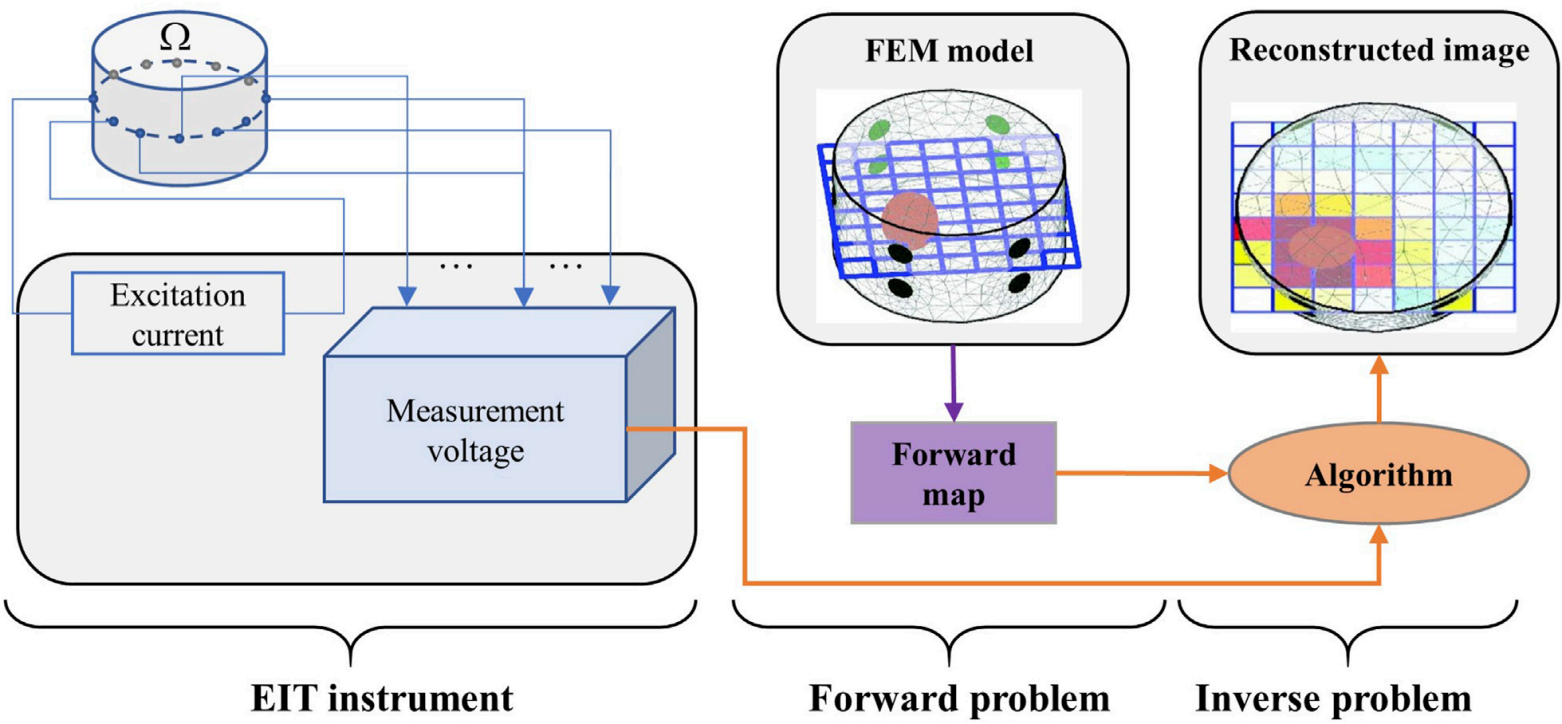
Typical schematic of image reconstruction. Left part: EIT measuring data from body Ω . Middle part: EIT forward problem for a finite element model of the body. Right part: EIT inverse problem, where forward mapping and reconstruction algorithms are used to reconstruct the EIT image from measured data (Lionheart and Adler, 2021).

EIT is typically divided into forward and inverse problem. The former pertains to calculating the surface voltage change based on conductivity distribution of the target body and the excitation current. The frequency of the EIT excitation current generally used for medical imaging is in the range of 10–100 kHz. In this frequency range, the influence of the dielectric properties can be ignored and the current field is treated as a steady-state field. As shown in Figure 1, we set the imaging domain as Ω . Based on Maxwell’s equations, the conductivity and potential distributions satisfy the following Laplace equation with natural boundary conditions.

$$\left\{\begin{array}{l} \nabla \cdot \left(\sigma \left(r\right)\nabla \Phi \left(r\right)\right)=0,\;r\in \Omega \\ \sigma \left(r\right)\nabla \Phi \left(r\right)\cdot n=J,\;r\in \partial \Omega \end{array}\right.$$ (1)

where the internal conductivity σ is a function of the spatial variable_;_ $\Phi \left(r\right)$ represents the potential at r within the field; $\bar{\Phi }$ represents the potential on a given boundary; n is the direction outside the normal; and J represents the current density of the injected excitation current at the boundary. The EIT forward problem can be addressed using the finite element method (FEM).

Generally, in practical EIT applications, the conductivity distribution inside the target body is unknown, and only the excitation current and measurement voltage at electrodes are known. Furthermore, as measurement errors are unavoidable, the observational model of EIT can be described as follows.

$$V=F\left(\sigma \right)+e$$ (2)

where V is the measured voltage; $F\left(\cdot \right)$ is the non-linear mapping between the conductivity distribution and boundary voltage, also known as the forward map; and e is the noise or measurement error.

The inverse problem of EIT, also known as image reconstruction, refers to reversely solving the conductivity distribution σ in the target domain according to the obtained measured voltage V . It is severely ill-posed, non-linear and ill-conditioned. Therefore, to obtain stable and fast EIT imaging with high resolution, researchers have proposed a number of image reconstruction methods, which can be divided into statistical inversion methods and deterministic methods. Reconstruction method based on the former are largely based on Bayesian theory, and use maximum likelihood estimation to iteratively solve the conductivity distribution when the maximum probability event meets the boundary conditions (Liu et al., 2018; Liu et al., 2020; Liu et al., 2021). In contrast, reconstruction methods based on the latter can be grouped into linear methods and non-linear reconstruction methods. Linear approximation methods primarily include the back-projection algorithm (Santosa and Vogelius, 1990), sensitivity matrix method (Morucci et al., 1994), Calderon method (Cheney et al., 1990), Newton’s one-step error reconstructor (NOSER) method (Le Hyaric and Pidcock, 2000), and GREIT algorithm in lung EIT (Adler et al., 2009). Whereas non-linear reconstruction methods primarily include non-linear optimization methods, which refer to global and local optimization search methods, and direct methods, which refer to the D-bar method (Hamilton et al., 2018; Hamilton et al., 2019b; Santos et al., 2020). In addition, based on shape constraints, scholars have proposed parameter set methods (Liu et al., 2018; Liu et al., 2019c; Liu et al., 2020a; Liu et al., 2020c) and shape reconstruction (Liu et al., 2019b; 2020b; Liu et al., 2020d; Gu et al., 2021a; Liu et al., 2021; Gu et al., 2021b; Liu and Du, 2021). With the tremendous advancement of artificial intelligence, machine learning methods are gradually being used in EIT image reconstruction and have achieved good reconstruction results.

## 3 Deep learning in EIT image reconstruction

A neural network is an artificial computing model that imitates the structure and function of animals’ nervous system. It is composed of multiple neurons and can model the complex relationship between data. With the rapid progress of deep learning, imaging with DNNs provide a powerful framework for EIT image reconstruction. This section highlights the application of deep learning in different ways of EIT image reconstruction and reviews three aspects: reconstruction of conductivity distribution directly from measurement data from a single neural network, joint reconstruction of traditional algorithms and deep learning, and hybrid reconstruction of multiple neural networks.

### 3.1 Single neural network-based direct reconstruction

#### 3.1.1 EIT reconstruction based on conventional neural network

In the early stage of neural network development, Guardo et al. proposed an EIT reconstruction technique using the adaptive linear element (ADALINE) neural network. They used the trained network structure to directly correlate the measurement data with the conductivity distribution; thus, solving the Jacobian matrix was not necessary (Guardo et al., 1991). Based on that, Adler and Guardo proposed a circular finite element model for numerical simulation to quickly generate a training set to solve the problem of time-consuming generation of training sets. The ADALINE network was trained without and with noise; the results revealed that training the network with noise exhibited better anti-noise performance and the imaging resolution was better as compared with the potential back-projection method (Adler and Guardo, 1994). Subsequently, researchers successively proposed methods based on pattern recognition, back-propagation networks, artificial neural networks (ANNs), and Bayesian multilayer perceptrons to solve the EIT inverse problem (Mikhailova et al., 1997; Nejatali and Ciric, 1998; Ratajewicz-Mikolajczak et al., 1998; Lampinen et al., 1999). However, these studies focused primarily on EIT reconstruction through training linear reconstruction operators and measured voltage, but do not focus on the non-linearity of EIT.

**FIGURE 2:**
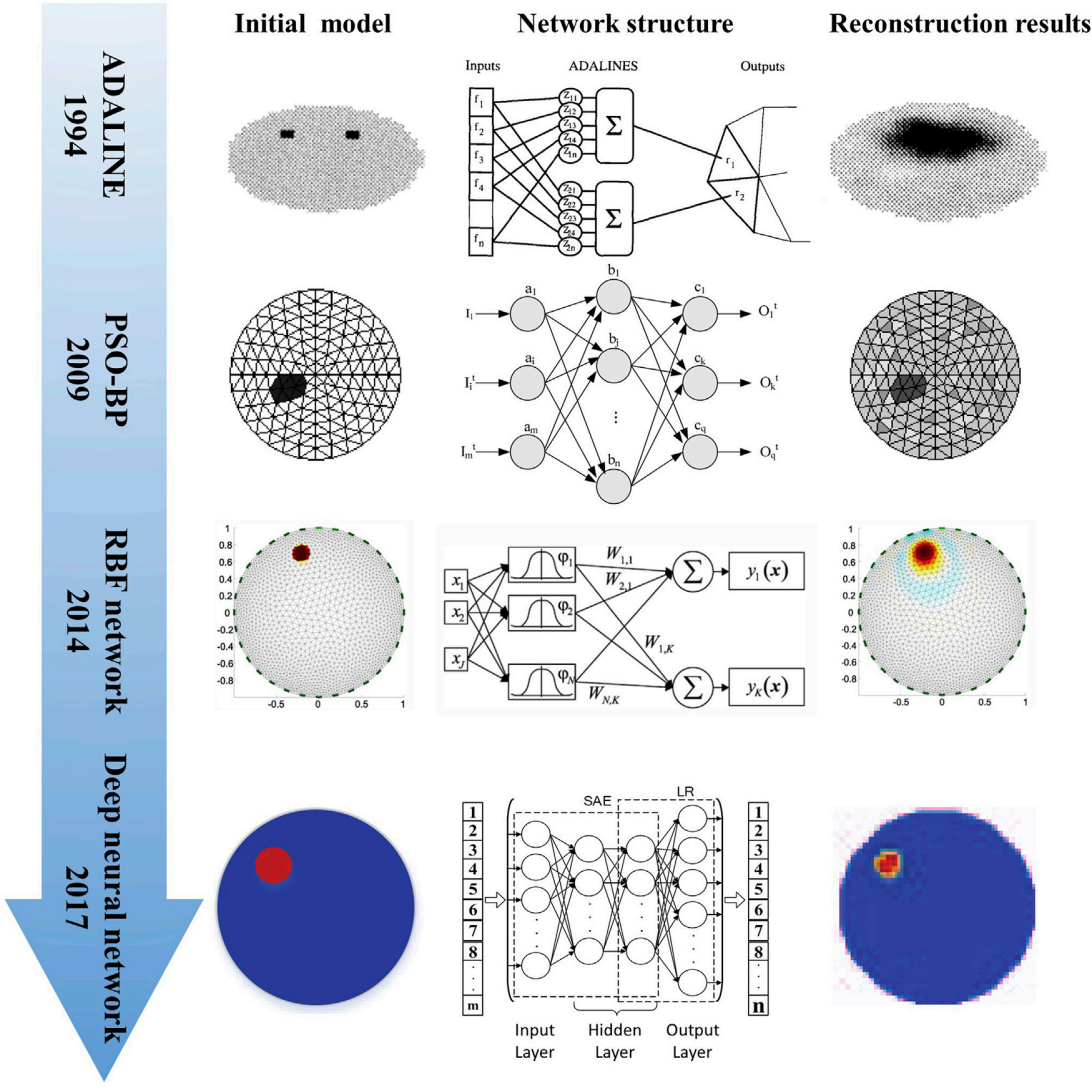
Progress in EIT reconstruction based on conventional neural networks (Adler and Guardo, 1994; Wang et al., 2009a; b; Michalikova et al., 2014; Li et al., 2017).

Considering the non-linearity of the EIT inverse problem, feed-forward neural networks with non-linear transfer functions were proposed to solve the EIT imaging problem (Acharya and Taylor, 2004). Wang et al. proposed a neural network based on the radial basis function (RBF) to construct a non-linear mapping model for EIT. Additionally, they optimized the parameters in the RBF network using a genetic algorithm; this resulted in a significantly better spatial resolution of imaging than the back-projection method (Wang et al., 2004). To further verify the feasibility of using the RBF neural network for reconstruction in EIT, Michalikova et al. used EIDORS to generate a 32-electrode simulation data set, and built and trained the RBF neural network. Their RBF neural network had 928 measured voltage inputs (input layer), 3214 conductivity distribution outputs (output layer), and could obtain imaging results similar to EIDORS (Michalikova et al., 2014). On this basis, Venclikova et al. further optimized the RBF neural network expansion factor (Venclikova et al., 2016). In regard with the RBF neural network problems of slow convergence and being prone to fall into the local minima, Zhang et al. proposed a method based on an algebraic neural network for EIT reconstruction and verified the performance of the algorithm *via* simulation (Zhang et al., 2009). Figure 2 shows the progress in EIT reconstruction based on conventional neural networks.

In the field of medical image reconstruction, because a DNN has stronger non-linear reconstruction ability compared with a shallow neural network, researchers have gradually begun to use multi-layer neural networks for EIT reconstruction. Li et al. proposed a four-layer DNN framework based on stacked autoencoders (SAEs) to build a non-linear mapping between measured voltage and internal conductivity; they verified the advantages of this method by simulation and phantom experiments (Li et al., 2017). Similarly, Zhang et al. (2021) proposed a four-layer DNN framework named EIT-4LDNN for EIT reconstruction. To obtain the accurate conductivity distribution in the target body, another previous study proposed a multi-layer ANN to solve the EIT inverse problem and subsequently reconstructed the conductivity distribution (Fernández-Fuentes et al., 2018). To determine the optimal ANN architecture and hyperparameter for the EIT inverse problem, Huuhtanen et al. investigated the effect of the width and depth of the multilayer perceptron on imaging quality (Huuhtanen and Jung, 2020). In addition, other researchers proposed a series of particle swarm optimization algorithms to optimize the network parameters to increase the convergence speed of the neural network in the training phase (Wang et al., 2009b; a; Martin and Choi, 2016).

#### 3.1.2 EIT reconstruction based on convolutional neural network

A convolutional neural network (CNN) is a type of neural network with convolution estimation and deep structure. It is a representative algorithm of deep learning and has been widely applied in numerous fields. In electromagnetic imaging, Tan et al. applied a deep learning method based on CNN to solve the image reconstruction of electrical resistance tomography. They utilized two convolutional layers to extract the key features in the voltage measurement and two pooling layers to shrink the network’s parameters. Additionally, to address the optimization problem in the initial model, the dropout layer and moving average method were applied, which significantly enhanced the system’s generalizability and training speed (Tan et al., 2019). Subsequently, to improve the quality of reconstruction results of EIT, Gao et al. (2019) proposed an EIT image reconstruction algorithm based on a convolutional denoising autoencoder; their method used a CNN in the encoder and decoder networks. In contrast to the traditional GREIT method, their proposed model does not require background calibration, reduces noise artifacts, and sharpens the boundaries of the imaging target. Compared with traditional SAEs and non-linear algorithms, this method is more robust.

Considering that the voltage signal collected by the system corresponds to one-dimensional data, converting the one-dimensional samples to two-dimensional samples is very time-consuming; additionally, the structure of the original measurement signal is possibly damaged, which may extract incorrect features from the two-dimensional signal. Li et al. proposed a one-dimensional CNN (1D-CNN) based on convolutional, pooling and fully connected layers to solve the direct reconstruction of EIT. In comparison with traditional DNNs and two-dimensional CNNs, simulation and physical model experiments revealed that this method has better edge retention, and anti-noise and generalization abilities, which confirmed its usefulness (Li X. et al., 2020).

**FIGURE 3:**
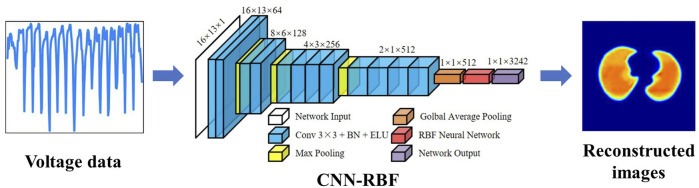
EIT image reconstruction of conductivity distribution directly from boundary voltage data using CNN (Wu et al., 2021).

Recently, Wu et al. optimized the CNN method based on the visual geometry group (VGG) model for lung EIT imaging by adding a batch normalization (BN) layer, ELU activation function, RBF network, and global average pooling (GAP) layer (Wu et al., 2021). The method directly learns the non-linear mapping between measurement voltage and conductivity distribution in an end-to-end manner. In comparison with the experimental results of the traditional Tikhonov, CNN and CNN-GAP algorithms, the simulation and experimental results revealed the robustness and effectiveness of the improved CNN-RBF. The network implementation process is shown in Figure 3.

### 3.2 Joint reconstruction of traditional EIT algorithm and deep learning

Although an ANN can be used to solve the EIT inverse problem, owing to the complexity of real clinical scenarios, obtaining good reconstruction results from actual data using simple ANNs is difficult. To overcome this issue, Martin et al. proposed a joint reconstruction method that first used the one step linear Gauss–Newton method to initially solve the EIT inverse problem and subsequently used an ANN to post-process the conductivity distribution (Martin and Choi, 2017). The benefits of linear and non-linear methods are combined in this strategy. In comparison with the one step Gauss–Newton, primal-dual interior-point, and pure ANN direct reconstruction methods, the results revealed that the proposed method has better stability and accuracy. Inspired by this, Dumdum et al. proposed a joint reconstruction strategy that first used the one step Gauss–Newton method to solve the EIT inverse problem and subsequently used the U-Net to post-process the image (Dumdum et al., 2019). In a recent work, Wang et al. used the NOSER method to obtain preliminary reconstruction and subsequently proposed a MobileNet-based PSPNet for post-processing the imaging results (Wang et al., 2021).

Lin et al. (2020) used a supervised descent method with flexible fusion of prior information and good generalization performance combined with a neural network with strong non-linear fitting ability for EIT image reconstruction. They proposed a neural network based on the supervised descent method (NN-SDM), which has the advantages of supervised descent method and neural network. They compared this joint reconstruction method with linear supervised descent, end-to-end neural network, and Gauss–Newton methods; the results revealed that the proposed method has a faster convergence speed and better generalization performance among the three methods.

**FIGURE 4:**
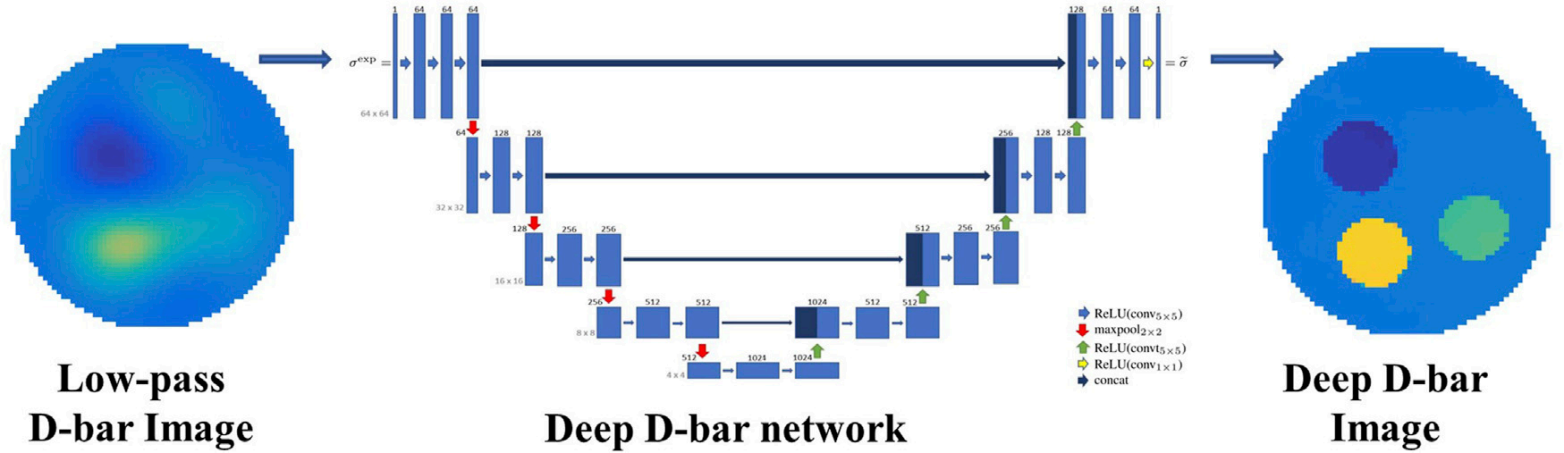
Deep D-Bar uses the initial low quality conductivity image obtained by traditional D-Bar and subsequently combines the U-Net to post-process the image and obtain a high quality static EIT image (Hamilton et al., 2018).

To overcome the issues of low image spatial resolution and additional under sampling artifacts caused by low-pass filtering in the traditional D-bar algorithm, Hamilton et al. (2018) proposed a deep D-bar method to reconstruct EIT images. As illustrated in Figure 4, the method first uses the D-bar algorithm to obtain the initial low-quality conductivity distribution image and then combines the U-Net to post-process the image to remove artifacts, thereby obtaining a high quality static EIT image with low time delay. Inspired by the deep D-bar, Hamilton et al. subsequently proposed a domain-independent Beltrami-net for EIT absolute imaging, which uses training data from the associated non-physical Beltrami equation instead of simulating the traditional current and voltage data specific to a given domain; this makes the training data independent of the shape of domain boundaries (Hamilton et al., 2019a). The results revealed that the suggested strategy is more resilient to border shape changes. Furthermore, in response to the problem of blurred internal organ boundaries in the reconstructed images generated by the traditional D-bar algorithm, Capps et al. proposed a method for sparse reconstruction that fused the reconstruction results of the normal D-bar algorithm with organ boundaries reconstructed by a neural network (Capps and Mueller, 2021). The method first uses D-Bar to obtain the reconstruction result, and subsequently, the organ boundaries are derived from the scattering transformation using deep learning methods. Finally, by fusing the normal D-bar reconstruction results with those of the neural network-reconstructed organ boundaries, the high-precision EIT reconstruction results of the organ boundaries are obtained.

**FIGURE 5:**
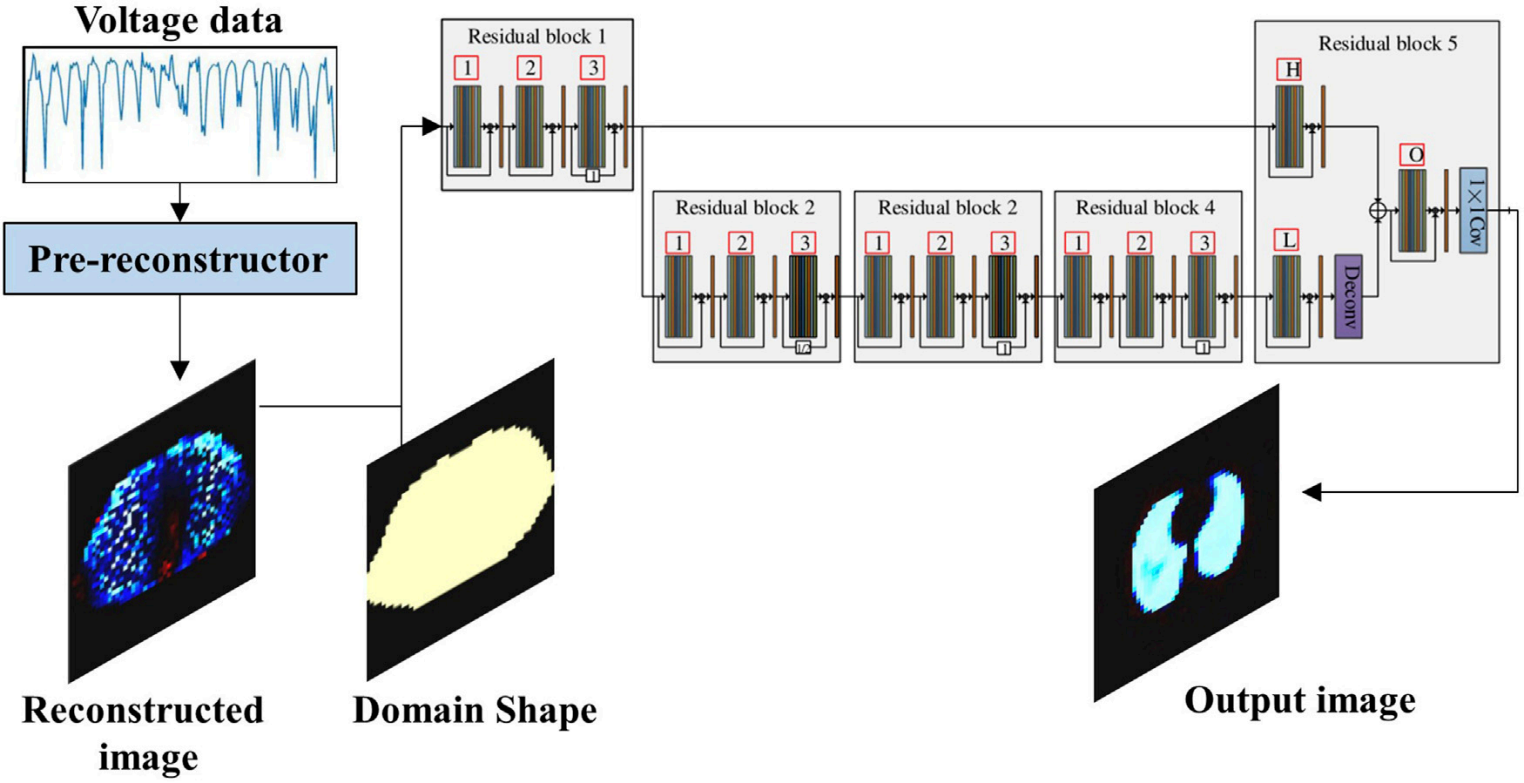
Two-stage deep learning method utilizing a CNN to post-process the pre-reconstruction (Ren et al., 2020).

In the traditional EIT reconstruction method, the regularization parameter has considerable influence on the imaging quality; thus, choosing an optimal regularization parameter is highly challenging. Aiming at this problem, a two-stage deep learning method was proposed by Ren et al. (2020); the method consists of a pre-reconstruction block and a CNN, as shown in Figure 5. In this method, the pre-reconstruction block learns regularization patterns from the training dataset and gives a crude reconstruction of the target. To eliminate modeling errors, a CNN post-processes the pre-reconstruction results through a multi-level feature analysis technique. The experimental results revealed that this method exhibited better reconstruction accuracy and robustness to noise compared with traditional algorithms, such as NOSER and total variation regularization. Zhang et al. (2022) proposed a network that employs a deep CNN to post-process the initial reconstruction of the conjugate gradient algorithm, called the V-shaped dense noise reduction network (VDD-Net). This method reduces the dependence on the exact forward mode, and in addition, the initial prior information allows the reconstruction of EIT images with high spatial resolution.

As described previously, CNNs are particularly beneficial in imaging applications due to their translational invariance and ability to exploit local dependencies and structures. However, for non-linear EIT problems, their forward models are usually discretized using triangular elements and solved by FME; therefore, it is necessary to convert the triangular mesh data into pixel grid data using an interpolation or equivalence step in order to apply CNNs to imaging tasks. To overcome this problem, Herzberg et al. proposed a flexible iterative Graph Convolution Newton-type Method (GCNM), which is capable of learning task-specific priors from training data using current iteration information and Newton-type update information and improves robustness to noise and model adaptation (Herzberg et al., 2021). The robustness of GCNM in terms of modeling errors is expected to provide a method to address the application research of EIT absolute imaging. For multi-frequency EIT in cell imaging, Chen et al. (2022a) proposed a mask-guided spatial-temporal graph neural network (M-STGNN) to simultaneously capture spatial and frequency correlations. Simulations and experiments showed that the M-STGNN achieves significant improvements in terms of both shape preservation and noise reduction compared with the state-of-the-art mfEIT image reconstruction algorithm. Exploiting the frequency and spatial correlation is an impressive technique for improving the image quality of multi-frequency electromagnetic tomography (Xiang et al., 2020). Based on this ideology, Chen et al. (2022b) proposed a multiple measurement vector network (MMV-Net) that integrated the advantages of the traditional Alternating Direction Method of Multipliers for the MMV problem (MMV-ADMM) and deep learning. By adding a spatial self-attention module and a convolutional long short-term memory module, which can adequately capture the intra-frequency and inter-frequency dependencies, it enhances picture quality, generalization ability, noise robustness, and convergence performance.

### 3.3 Hybrid deep learning reconstruction for EIT

In addition to the joint reconstruction using deep learning and traditional algorithms, hybrid deep learning reconstruction for EIT is a popular way of deep learning in EIT reconstruction. Hrabuska et al. (2018) reported that first using radial basis neural network reconstruction and subsequently using a Hopfield neural network to filter the image will obtain better reconstruction results through simulation experiments. Subsequently, Huang et al. (2019) proposed a method combining an ANN and U-Net for EIT image reconstruction. The method first uses the adaptive moment estimation optimization algorithm and mean square error function to train an ANN for reconstructing the initial EIT image, and then uses U-Net for image post-processing to obtain higher quality EIT images (Huang et al., 2019).

Considering the strong correlation between the measured values of some electrodes in the EIT measurement, Rymarczyk et al. proposed a hybrid reconstruction method to reduce the computational time and achieve fast imaging (Rymarczyk et al., 2018). The method first uses ElasticNet to remove the relevant prediction vectors and then trains an ANN to obtain the reconstruction results. The hybrid algorithm speeds up the neural network training and image reconstruction process, thus rendering the system more robust to the noise of input data. With a similar purpose, Chen et al. were inspired by the concept of transfer learning and proposed a hybrid reconstruction method for EIT called FC-UNet. This method first inputs the measured voltage data into a simple network that only contains fully connected and ReLU layers to generate an initial image, and subsequently uses U-Net to denoise the image to obtain the final EIT reconstructed image (Chen et al., 2020). In order to address the challenge of accurately reconstructing continuous, multilevel conductivity distributions in multiple objects settings *via* EIT in tissue engineering applications, Chen et al. (2021) proposed a deep learning and group sparsity (DL-GS) regularization-based hybrid algorithm for miniature EIT on the architecture of FC-UNet. The method estimates structural information using deep neural networks and then estimates continuous conductivity distributions using group sparsity regularization. Following that work, they proposed a structure-aware two-branch network (SADB-Net) that fuses information together by two feature extractors, and the results showed that SADB-Net can obtain high-quality reconstructed images at multi-target, multilevel conductivity distributions, which can be well applied to dynamic cell culture for tissue engineering (Chen and Yang, 2021). Different from the study by (Chen Z. et al., 2020), Ye et al. (2021) proposed to expand the data only through the splicing layer and subsequently input it into the U^2^-Net network to realize a hybrid reconstruction method called CAT + U2-Net. In addition, they have also recently proposed a 3D reconstruction method for composite electrode EIT systems using U^2^-Net (Ye et al., 2022).

**FIGURE 6:**
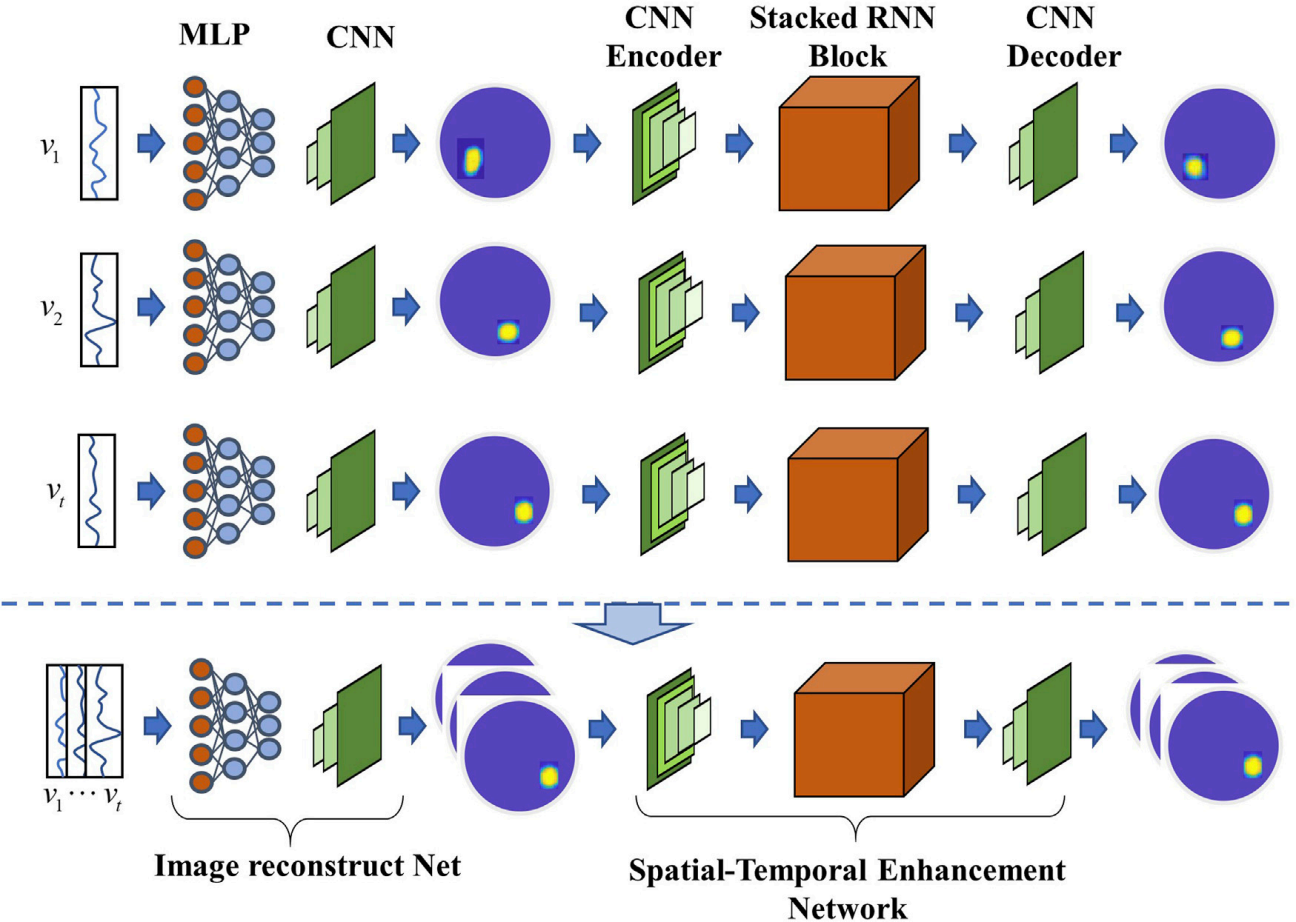
Typical hybrid deep learning reconstruction network RCRC that can automatically learn prior spatial-temporal information from the training dataset and utilize it to enhance the conductivity reconstruction accuracy (Ren et al., 2021).

Owing to the severe ill-posedness of the EIT inverse problem, Seo et al. (2019) suggested an image reconstruction method based on manifold learning to transform it into a well-posed one, and introduced its application in lung time-difference EIT imaging. This method first uses a variational autoencoder to identify the low-dimensional latent space encoding of useful lung images, subsequently learns the non-linear regression map between EIT measurement data and low-dimensional latent variables, and finally performs image reconstruction (Seo et al., 2019; Ko and Cheng, 2021). Fan et al. proposed a novel neural network architecture for the EIT problem, which combined a 2D CNN based on BCR-Net for EIT image reconstruction (Fan and Ying, 2020). Considering that the measured voltages or target images in EIT dynamic imaging are spatiotemporally correlated, Ren et al. (2021) proposed a RCRC DNN, comprising a reconstruction network, recurrent neural network model, CNN encoder, and CNN decoder, as shown in Figure 6.

## 4 Discussion

In this review, we systematically analyzed the application and development of deep learning technology in EIT image reconstruction from three aspects: neural network reconstruction directly from EIT measurement data, traditional algorithm and deep learning joint reconstruction, and multiple network hybrid reconstruction. A summary of the application of deep learning in EIT reconstruction and analysis is presented in Table 1. It should be noted that most of the computer configurations currently used for deep learning tasks listed in Table 1 are relatively high-end. However, for most of the methods, once the model is trained, a properly configured personal computer can also perform EIT reconstruction quickly.

**TABLE 1:** Summary of the deep learning application in EIT reconstruction and analysis.

Category	Model	Main Methods	Applications	Metrics for improvement	Computer Configuration	References
Single neural network-based direct reconstruction	CNN	Based on LeNet and refined by dropout layer and moving average	To solve the EIT inverse problem	Lower relative image error (RIE) Higher Image correlation coefficient (ICC)	GPU: Nvidia TITAN Xp	Tan et al. (2019)
	CNN-RBF	Based on VGG model by adding a BN layer, ELU activation function, RBF network, and GAP layer	High-resolution and robust shape reconstructions with multiphase conductivity for EIT lung imaging	Lower root mean square error (RMSE); Higher ICC	GPU: Nvidia GeForce GTX 1660 RAM: 16 GB	Wu et al. (2021)
	1D-CNN	Utilizes the convolutional layer, pooling layer, and full connection layer	To solve the EIT inverse problem; Industrial Process Tomography	Lower RIE and higher ICC	Requires a GPU, model unknown	Li et al. (2020b)
Joint reconstruction of traditional EIT algorithm and deep learning	One-stepGN+ ANN	Applying the ANN after the linear one-step GN	To solve 3D EIT problems	Lower position error(PE) and shape deformation (SD)	CPU: Intel Core I7-6700 RAM: 64 GB	Martin and Choi (2017)
	Beltrami-net	Pairs deep learning with D-bar methods and examine the effect of prior information	For absolute imaging with EIT	Higher structural similarity indices (SSIM); Lower RIE	GPU: Nvidia Titan XP	Hamilton et al. (2019a)
	Deep D-bar	Fuses the results of the output of the CNN with reconstruction computed by the D-bar method	Reconstruction of organ boundaries for lung EIT	Higher SSIM boundaries and image	GPU: Nvidia RTX 2080	Hamilton et al. (2019b); Capps and Mueller (2021)
	TSDL	Consists of the pre-reconstruction block to learn regularization patterns and a CNN to perform post-processes in a multi-level feature analysis strategy	For robust shape reconstruction of the lung	Lower RMSE Higher SSIM	CPU: Intel Xeon E5-2630 GPU: Nvidia GTX Titan X RAM: 64 GB	Ren et al. (2020)
	GCNM	Interprets the mesh as a graph and formulates the network using a graph convolutional neural network	For nonlinear inverse problem, such as EIT	Lower mean squared error (MSE) and more robust	GPU: Nvidia Titan V	Herzberg et al. (2021)
	M-STGNN	Simultaneously captures spatial and frequency correlations	mfEIT image reconstruction	Higher peak signal-to-noise ratio (PSNR) and SSIM; Lower RMSE	GPU: Nvidia P5000	Chen et al. (2022b)
	VDD-Net	Employs a deep CNN to post-process the initial reconstruction of the conjugate gradient algorithm	To obtain high spatial resolution EIT images of the lungs	Lower reconstruction error, distortion and widening; Higher SSIM	GPU: Nvidia RTX 2080Ti	Zhang et al. (2022)
	MMV-Net	Integrates the advantages of MMV-ADMM and deep learning	mfEIT image reconstruction	Higher peak signal-to-noise ratio (PSNR) and SSIM Lower RMSE	GPU: Nvidia P5000	Chen et al. (2022b)
Hybrid deep learning reconstruction for EIT	VAE+encoder-decoder	Utilizes manifold learning to transform EIT inverse problem into a well-posed one	Lung time-difference EIT imaging	Higher image reconstruction quality	GPU: Nvidia GeForce GTX 1080 Ti	Seo et al. (2019)
	ANN+U-Net	RBF networks reconstructs initial image and U-Net is used for post-processing	To solve the inverse problem and post-processing images	Higher resolution Lower PE	GPU: Nvidia GeForce GTX 1080 Ti	Huang et al. (2019)
	RCRC	Includes a reconstruction network, recurrent neural network, and CNN encoder and decoder	Dynamic image reconstruction	Lower RMSE Higher SSIM	CPU: Intel(R) Xeon(R) E5-2630; RAM: 64 GB; GPU: Nvidia Titia Xp	Ren et al. (2021)
	DL-GS	FC-UNet estimates the structural information and GS regularization obtains the final results	Cell imaging	Higher CC and better image quality	GPU: Nvidia P5000	Chen et al. (2021)
	FCN+U2-Net	The input layer is Full Connection Network (FCN) and the backbone is U2-Net	3D EIT imaging for tumor boundary detection	Higher mean SSIM and better quality of 3D imaging	Not mentioned	Ye et al. (2022)
	SADB-Net	Two independent branches to encode the structure and conductivity features	Cell imaging of multi-object, multi-value conductivity distributions	Lower RIE	GPU: Two Nvidia P5000s	Chen and Yang (2021)

In general, with the ongoing advancement of deep learning, EIT image reconstruction based on deep learning can often obtain better imaging results, compared with traditional EIT reconstruction methods. However, most of the current research is limited to simulation and phantom experiments. There are still some challenges remaining for the future advancement of deep learning-based EIT to practical clinical and industrial applications. 1) Training a good deep learning model requires a large amount of data, which is time-consuming and laborious to obtain, especially for medical applications where human data is more difficult to obtain. 2) Overfitting is a common issue with deep learning models, which leads to a significant reduction in generalizability in practical applications. Although there are ways to mitigate this, it is still a non-negligible problem for practical applications. 3) Deep learning involves substantial programming knowledge, adjusting of parameters, and bug-fixing abilities, all of which might be challenging for beginners utilizing deep learning-based EIT. Some of the following directions may be something we may work on in the future in order to gradually transition deep learning-based EIT to real-world applications.

### 4.1 High quality EIT dataset for deep learning

A deep learning model’s capacity for learning is mostly determined by the training dataset. Most current datasets are generated based on 2D or 3D simulation models, and some differences with the actual EIT data obtained from the human body still exist. The measurement noise of a hardware system in a real environment is typically irregular, whereas the noise added to the training data in a simulation is typically of a known distribution. In addition, owing to the complexity of the actual hardware system, accurately modeling the hardware system is very difficult. Therefore, datasets based on simulation models often cannot accurately reflect true EIT measurements. Gaggero et al. (2014) initially explored the possibility of using real saltwater tank model data for training. However, this dataset only contained training data of 770 different locations, and the total amount of data was small. In contrast, owing to the data collection patterns of different EIT systems, the current way of increasing data sets by sharing data among research groups has some difficulties. As deep learning requires a considerable amount of high quality training data (Sun et al., 2017), numerous studies on extending an EIT dataset based on variational autoencoders and generative adversarial networks (Chen et al., 2020; Zhan et al., 2021) have been conducted. Non-etheless, building high quality EIT datasets for training is still a major problem, which can be solved collaboratively by EIT research groups.

### 4.2 Building efficient deep learning models in EIT

As the data set used for training is always limited, if deep learning is only used as a black-box solver to directly learn the mapping relationship between the measured voltage signal and output conductivity distribution, the training results may have lower generalization ability under the training of limited sample data. Therefore, the method of reducing the dependence of deep learning on datasets is also an issue that needs to be considered. One possible way, as described in Section 3.2, is utilizing traditional algorithms combined with deep learning for EIT image reconstruction. Traditional algorithms are based on well-established mathematical and physical principles, and generate outputs corresponding to their inputs in a fixed manner, regardless of generalization issues. If the prior knowledge of physics and mathematics in traditional algorithms can be integrated into the deep learning network, the non-linearity of the neural network mapping function can be reduced and the generalization ability of the model can be improved. In addition, the optimization of traditional imaging methods is worth studying. For example, based on the concept of induced contrast current, Wei et al. (2019) proposed a basis-expansion subspace optimization method to solve the inverse problem of EIT and a deep learning method based on dominant current; this improved the generalization of the network and enabled fast, high quality and stable EIT imaging.

In addition to requiring considerable training data, deep learning consumes considerable computing resources. To obtain strong representation ability, the typical deep learning models must require a significant number of parameters. Subsequently, training and testing these models require more memory and computing power. For example, the U-Net network, a popular tool for medical imaging, requires hours of training time on a Nvidia Titan GPU (6 GB) although the computational overhead has been minimized (Ronneberger et al., 2015). This poses some challenges to the portability of EIT hardware systems. Therefore, optimizing the network structure to reduce hardware resource requirements should be considered in future deep learning technology for EIT. Recently, Alford et al. proposed a pruned and structurally sparse neural network (Alford et al., 2018), and Hosseini et al. proposed a recurrent sparse connection architecture (Hosseini et al., 2021); these studies provide a new research direction for deep learning-based EIT image reconstruction as well as a novel perspective for the development of intelligent portable EIT.

### 4.3 Smarter multi-modality EIT and image fusion

As EIT is a type of functional imaging, its poor spatial resolution is a major issue in realizing its clinical application. Therefore, the main task of the current deep learning-based EIT image reconstruction technique is to improve the reconstruction quality and spatial resolution of imaging. Multi-modality imaging is used to improve the image quality of EIT; for example, dual-modality imaging of EIT and ultrasound based on acousto-electric effect significantly improve the image quality of imaging results (Liang et al., 2017; Liu et al., 2019). Recently, Liu et al. reported an impedance optics-dual-modal imaging framework for 3D cell culture imaging, where they used a multiscale feature cross-fusion network (MSFCF-Net) to fuse the information between different modalities. In addition, Liu et al. (2022a) also proposed a multimodal reconstruction algorithm based on the Kernel method that originated from machine learning and obtained excellent EIT images (Liu and Yang, 2022). In comparison with traditional methods, multi-modality learning based on deep learning has several advantages. Ramachandram et al. reviewed the development of deep multi-modality learning in existing literature (Ramachandram and Taylor, 2017), which provides a new development idea for future multi-modality imaging of EIT.

In addition, using CT images to assist EIT imaging and encoding the structural information in CT images in the regularization term to constrain the conductivity estimation is another method to improve the imaging quality of EIT (Li et al., 2020). This type of image fusion technology has been initially developed by Xu et al. to fuse CT and EIT images for obtaining EIT-CT images, thereby providing doctors with more intuitive diagnostic information (Xu et al., 2011). However, this method is limited to registration of images and does not realize the utilization of CT data information. A similar approach was applied in the study by (Reinartz et al., 2019) to provide real-time ventilation image information for the lungs. In comparison with traditional methods, deep learning has achieved better results in data information utilization and medical image registration; this also provides a new technical means for improving the imaging quality of EIT through image fusion technology.

### 4.4 3D EIT

Because the propagation of current in space is not confined to the 2D electrode plane but in 3D space, the EIT essentially reflects the conductivity distribution in 3D space. However, most of the current reconstruction algorithms on EIT focus on 2D, even for deep learning-based reconstruction methods. When the 2D approach is extended to 3D, it leads to a significant increase in the number of dimensions, requires more computational resources, and makes it difficult to train the model efficiently so that it can be reconstructed accurately. In order to solve the non-linear 3DEIT inverse problem, Martin et al. proposed a solution based on the divide-and-conquer method and ANNs (Martin and Choi, 2018). This solution caps the number of outputs for each individual ANN and subsequently lowers the number of weights and biases in each individual ANN, greatly accelerating training and enhancing global convergence. Yi et al. (2022) proposed a transposed convolution with neurons network (TN-Net) to solve the image reconstruction problem of 3D EIT. The DNN method proposed by Fan et al. can be used for both 2D and 3D imaging of EIT (Fan and Ying, 2020). In addition, for the needs of 3D cell culture process monitoring, researchers have also proposed numerous deep learning-based methods that could be extended to 3D, such as SADB-Net (Chen and Yang, 2021), GCNM (Herzberg et al., 2021), MSFCF-Net (Liu et al., 2022a), M-STGNN (Chen et al., 2022a),MMV-Net (Chen et al., 2022b), *etc.*, which provide a large number of algorithmic bases for future 3DEIT reconstruction studies.

### 4.5 Intelligent medical decision-making based on EIT

Although this study primarily focus the advancement of deep learning in EIT image reconstruction, deep learning plays a significant role in solving other aspects of EIT. In particular, in terms of medical diagnosis and decision-making, deep learning can be used to provide doctors with intelligent auxiliary diagnosis information for quick decision-making. Candiani et al. used neural networks to achieve effective classification of brain stroke from EIT results (Candiani and Santacesaria, 2022), whereas Dunne et al. used image-based machine learning that provides intelligent monitoring for urinary incontinence patients (Dunne et al., 2018). Moreover, Lee et al. (2020) proposed EIT abdominal fat estimation based on deep learning. In addition, in lung EIT used in clinical research, researchers achieved the separation of cardiac images using the semi-Siamese U-Net (Ko and Cheng, 2021) to obtain cardiac impedance images that can be used for bedside diagnosis; this provides a new method and diagnostic basis for doctors. The aforementioned studies indicate that deep learning plays a significant role in medical diagnosis of EIT, which may facilitate the clinical application of EIT in future.

## 5 Conclusion

At present, deep learning plays an important role in EIT image reconstruction, and has had a significant impact on improving the quality of EIT reconstruction. The simple way of reconstructing EIT images directly from measurement data based on neural networks cannot meet the complex clinical use scenarios owing to insufficient generalization ability of the model. In future, the joint reconstruction method based on traditional reconstruction algorithms and deep learning, and the use of multiple networks for hybrid reconstruction will be the main development directions of deep learning in EIT image reconstruction. Currently, EIT image reconstruction based on deep learning still deals with certain problems in terms of datasets, and establishing shared datasets through the cooperation of more research teams is necessary. In addition, deep learning can be combined with traditional algorithms to design a better network structure to ensure that it can better integrate prior information, improve the generalization ability of the model, and expand the application prospects of EIT by integrating multi-modal intelligent imaging; some of them could be solutions to existing challenges.

In conclusion, deep learning provides a new method for EIT image reconstruction and to solve the problems faced by EIT in clinical settings. The successful application of deep learning in EIT image reconstruction has laid a foundation for the establishment of an intelligent integrated EIT diagnostic system in the future.
